# Systemic Therapy for Hepatocellular Carcinoma: Latest Advances

**DOI:** 10.3390/cancers10110412

**Published:** 2018-10-30

**Authors:** Masatoshi Kudo

**Affiliations:** Department of Gastroenterology and Hepatology, Faculty of Medicine, Kindai University, 337-2 Ohno-Higashi, Osaka-Sayama, Osaka 589-8511, Japan; m-kudo@med.kindai.ac.jp; Tel.: +81-72-366-0221; Fax: +81-72-367-2880

**Keywords:** hepatocellular carcinoma, systemic therapy, molecular targeted therapy, immune checkpoint inhibitor

## Abstract

Systemic therapy for hepatocellular carcinoma (HCC) has changed drastically since the introduction of the molecular targeted agent sorafenib in 2007. Although sorafenib expanded the treatment options for extrahepatic spread (EHS) and vascular invasion, making long-term survival of patients with advanced disease achievable to a certain extent, new molecular-targeted agents are being developed as alternatives to sorafenib due to shortcomings such as its low response rate and high toxicity. Every single one of the many drugs developed during the 10-year period from 2007 to 2016 was a failure. However, during the two-year period from 2017 through 2018, four drugs—regorafenib, lenvatinib, cabozantinib, and ramucirumab—emerged successfully from clinical trials in quick succession and became available for clinical use. The efficacy of combination therapy with transcatheter arterial chemoembolization (TACE) plus sorafenib was also first demonstrated in 2018. Recently, immune checkpoint inhibitors have been applied to HCC treatment and many phase III clinical trials are ongoing, not only on monotherapy with nivolumab, pembrolizumab, and tislelizumab, but also on combination therapy with checkpoint inhibitors, programmed death-1 (PD-1) or PD-ligand 1 (PD-L1) antibody plus a molecular targeted agent (bevacizumab) or the cytotoxic T-lymphocyte-associated antigen 4 (CTLA-4) antibody, tremelimumab. These combination therapies have shown higher response rates than PD-1/PD-L1 monotherapy alone, suggesting a synergistic effect by combination therapy in early phases; therefore, further results are eagerly awaited.

## 1. Introduction

Systemic therapy for hepatocellular carcinoma (HCC) has changed drastically since the introduction of the molecular targeted agent, sorafenib in 2007. Although sorafenib expanded the treatment options for extrahepatic spread (EHS) and vascular invasion, making long-term survival of patients with advanced disease achievable to a certain extent, new molecular targeted agents have been attempted to develop as alternatives to sorafenib due to shortcomings such as its low response rate and high toxicity. Every single one of the many drugs developed during the 10-year period from 2007 to 2016 was a failure [1]. However, during the two-year period from 2017 through 2018, four drugs—regorafenib, lenvatinib, cabozantinib, and ramucirumab—emerged successfully from clinical trials in quick succession and became available for clinical use. The efficacy of combination therapy with transcatheter arterial chemoembolization (TACE) plus sorafenib was also first demonstrated in 2018 [2].

This review describes the current landscape of molecular targeted therapy for HCC, challenges that remain to be solved, and potential future developments.

## 2. Molecular Targeted Agents

### 2.1. Sorafenib

Sorafenib is an oral kinase inhibitor that exerts its antitumor effects by suppressing tumor proliferation through inhibition of serine/threonine kinases of *C-Raf*, wild-type *B-Raf*, and mutant *B-Raf^V600E^*, which are components of the Raf/MEK/ERK pathway (MAP kinase pathway) downstream of vascular endothelial growth factor receptor (VEGFR), platelet-derived growth factor receptor ( PDGFR), and epithelial growth factor receptor (EGFR), as well as by suppressing angiogenesis through inhibition of tyrosine kinases such as VEGFR1, VEGFR2, VEGFR3, PDGFRβ, RET, and FLT-3 (fms-related tyrosine kinase-3) [3,4]. Sorafenib was shown to significantly prolong overall survival (OS) over placebo in two large trials (the SHARP trial and Asia-Pacific trial) [1,5] and has consequently become the standard therapy for advanced HCC.

### 2.2. Current Landscape of Molecular Targeted Drug Development for HCC

Several clinical trials of new molecular targeted drugs have been conducted to date [1]. The trials can be broadly classified into four categories: (1) adjuvant therapy after curative therapy, (2) combination therapy with TACE, (3) first-line therapy for advanced HCC, and (4) second-line therapy for advanced HCC. Results of phase III trials are described below.

#### 2.2.1. Prevention of Recurrence After Curative Therapy (Adjuvant Therapy)

Three phase III trials, one comparing vitamin K2 with placebo as adjuvant chemotherapy after radiofrequency ablation or resection [6], one comparing sorafenib with placebo (STORM trial) [7], one comparing peretinoin with placebo (NIK333 trial) [8], and one comparing ablation plus lyso-thermosensitive liposomal doxorubicin [9] have been conducted to date, but all of them failed (Table 1). However, an Asian trial of peretinoin in patients with HCC associated with hepatitis B is currently ongoing in Japan, South Korea, and Taiwan. A phase III trial comparing the anti-programmed death (PD)-1 antibody, nivolumab with placebo after curative therapy is also ongoing (Table 1).

#### 2.2.2. Combination Therapy with TACE

Three trials of sorafenib combination therapy with TACE, namely, a phase III trial in Japanese and Korean patients (Post-TACE trial) [10], a phase II trial comparing sorafenib plus TACE with drug-eluting beads (DEB-TACE) to placebo plus DEB-TACE (SPACE trial) [11], and a phase III trial also investigating sorafenib combination with DEB-TACE (TACE 2 trial) [14], have been conducted to date, but all of them failed due to not meeting the primary endpoints of prolonging time to progression (TTP) or progression-free survival (PFS). Phase III trials of the molecular targeted agents, brivanib and orantinib, in combination with TACE, were also conducted, but they also failed due to not meeting the primary endpoint of prolonging OS [12,13].

By learning the lessons from these five negative trials, the definition of “progression” for TACE trials as an endpoint was newly designed, better reflecting how TACE is performed in clinical practice. After application of this newly defined “progression”, results of the first positive trial to demonstrate the clinical efficacy of TACE plus sorafenib (TACTICS trial) were presented at the American Society of Clinical Oncology Gastrointestinal Cancers (ASCO-GI) Symposium in 2018 [2]. In the TACTICS trial, PFS was significantly longer with TACE plus sorafenib than with TACE alone (25.2 months vs. 13.5 months) [2].

#### 2.2.3. First-Line Therapy for Advanced HCC

##### Overview of First-Line Trials Conducted to Date

Head-to-head trials comparing sorafenib with single-agent sunitinib [15], brivanib [16], and linifanib [17] were conducted, but none of them was able to demonstrate superiority or non-inferiority to sorafenib. Phase III trials assessing the superiority of combination therapy with sorafenib plus erlotinib [18], doxorubicin, or hepatic arterial infusion chemotherapy (HAIC) [19,20] with an implanted reservoir system [21] compared with sorafenib alone all failed as well. Two head-to-head trials comparing radioembolization with Y90 to sorafenib also failed [22,23]. In summary, a total of eight first-line trials have failed to date [24] (Table 2).

##### Lenvatinib: Overview of REFLECT Trial Results

The REFLECT trial was the only trial with positive outcomes during this 10-year period of negative trials. Lenvatinib is an oral kinase inhibitor that selectively inhibits receptor tyrosine kinases involved in tumor angiogenesis and tumor growth (e.g., VEGFR1, VEGFR2, VEGFR3, fibroblast growth factor receptor (FGFR)1, FGFR2, FGFR3, FGFR4, PDGFRα, KIT, and RET) [34,35]. A single-arm phase II trial in advanced HCC showed excellent results (TTP: 7.4 months; OS: 18.7 months) [36]. The phase III REFLECT trial comparing sorafenib and lenvatinib was then conducted [25].

The REFLECT trial was a global phase III trial assessing the non-inferiority of lenvatinib to sorafenib. Patients were stratified by race (Asian or non-Asian), vascular invasion and/or EHS (yes or no), Eastern Cooperative Oncology Group performance status (PS) (0 or 1), and body weight (<60 kg or ≥60 kg). Treatment was continued until disease progression or onset of an intolerable adverse event (AE). Non-inferiority of OS was evaluated as the primary endpoint (non-inferiority margin = 1.08). Secondary endpoints are PFS, TTP, objective response rate (ORR), and safety.

Of the enrolled patients, 478 were assigned to the lenvatinib group and 476 to the sorafenib group. Body weight was less than 60 kg in 32% of patients and 60 kg or higher in 68%. Vascular invasion and/or EHS was present in 69% of patients. The number of patients with HCC due to hepatitis C was favorably imbalanced into the sorafenib group (27% vs. 19% in the lenvatinib group) [37]. Conversely, the number of patients with HCC due to hepatitis B was 53% in the lenvatinib group. An alpha-fetoprotein (AFP) level over 200 ng/mL was seen in the lenvatinib group more frequently than in sorafenib group (46% vs. 39%).

The primary endpoint of OS was 13.6 months in the lenvatinib group and 12.3 months in the sorafenib group, with a hazard ratio of 0.92 (0.79–1.06). The upper limit of the 95% confidence interval (CI) was below the prespecified non-inferiority margin of 1.08, which statistically showed a positive result; the non-inferiority of lenvatinib with respect to OS [25]. PFS (7.4 months in the lenvatinib arm vs. 3.7 months in the sorafenib arm), TTP (8.9 months vs. 3.7 months), and ORR (24.1% vs. 9.2%) per investigator using the modified RECIST criteria (mRECIST) were also better in the lenvatinib arm than the sorafenib arm, thus demonstrating the significantly better antitumor effect of lenvatinib [25]. Another surprising finding was that tumor shrinkage and necrotizing effect were excellent in the lenvatinib group as demonstrated by ORR per independent imaging review using mRECIST (40.6% in the lenvatinib vs. 12.4% in the sorafenib group) [25]. This favorable antitumor effect demonstrated by PFS, TTP, and ORR was also seen in independent imaging review using RECIST 1.1. [38].

Since patients were not stratified by AFP, a higher proportion of patients with AFP over 200 ng/mL were seen in the lenvatinib group than in the sorafenib group. When this AFP imbalance was corrected by covariate analysis, lenvatinib was statistically shown as superior to sorafenib with respect to OS (hazard ratio (HR) = 0.856, 95% CI 0.736–0.995, nominal *p*-value = 0.0342) [25,39]. This result suggests that this global trial could have shown superiority if AFP was included as a stratification factor.

In OS subanalysis, lenvatinib showed longer OS than sorafenib in almost all subgroups. One particularly important finding was that lenvatinib demonstrated longer OS than sorafenib even in patients with a body weight of less than 60 kg receiving a dose of only 8 mg, and the HR was similar to or slightly better than in patients with body weight of 60 kg or more who received 12 mg (<60 kg, HR = 0.85 vs. ≥60 kg, HR 0.95). This data suggest that weight-based dosing was successful. Longer OS was shown in patients with high baseline AFP (≥200 ng/mL), a poor prognostic factor, as revealed by the HR of 0.78 (95% CI, 0.63–0.98). Treatment duration was 5.7 months in the lenvatinib group and 3.7 months in the sorafenib group, indicating that patients were more tolerant to the lenvatinib.

The above results statistically demonstrate the non-inferiority of lenvatinib to sorafenib with respect to OS, and all the secondary endpoints (PFS, TTP, and ORR) showed statistically and clinically significant improvement as well. These findings demonstrated the efficacy of lenvatinib as a first-line agent for unresectable HCC. On 23 March 2018, HCC was aproned in Japan as another indication for lenvatinib along with the previously approved indication of thyroid cancer followed by the United States, Europe, China, and Korea.

#### 2.2.4. Second-Line Therapy for Advanced HCC

Sorafenib is the standard therapy for advanced stage HCC, so placebo-controlled comparative trials were conducted in patients who progressed on sorafenib or were intolerant to sorafenib and could not continue treatment due to adverse reactions.

#### Overview of Second-Line Trials Conducted to Date

A total of eight placebo-controlled trials of drugs such as brivanib [26], everolimus [27], ramucirumab [28], S-1 [29], arginine deiminase-conjugated with polyethylene glycol (ADI-PEG20) [30], and tivantinib [32] were conducted, but all of them failed (Table 2).

#### Regorafenib: Overview of the RESORCE Trial

Regorafenib is an oral multikinase inhibitor of protein kinases such as VEGFR1, VEGFR2, VEGFR3, TIE2, PDGFRβ, FGFR, KIT, RET, RAF-1, and BRAF [40]. Its molecular structure is nearly identical to that of sorafenib, which gives it a very similar toxicity profile. Unlike other drugs, it was investigated in a phase III placebo-controlled trial in patients refractory to sorafenib but not intolerant to sorafenib. The primary endpoint of OS was significantly better in the regorafenib arm than the placebo arm (10.6 months vs. 7.8 months) [31]. PFS and TTP were also significantly better. Regorafenib became the first drug demonstrated to show efficacy compared with placebo in second-line therapy. After these results were presented, HCC was added as an indication for regorafenib after progression on sorafenib in Japan in May2017. However, second-line therapy for sorafenib-intolerant patients remains an unmet need because this drug is generally not suitable for use in that population.

The key factor of success of the RESORCE trial can be attributed to the following four factors: (1) patients who discontinued sorafenib due to adverse reactions were excluded from the trial, leaving only patients with progressive disease (PD) on sorafenib, (2) imbalances between the active drug and placebo arms were avoided by including vascular invasion and EHS as separate stratification factors, (3) AFP was also included as a stratification factor, and (4) only patients with adequate tolerance to sorafenib (patients able to take at least 400 mg of sorafenib for at least 20 of the 28 days preceding the PD assessment) were included. This trial design prevented dropouts due to adverse reactions to regorafenib and minimized the effect of post-trial treatment after PD on regorafenib [31]. According to the results of the RESORCE trial, median survival time on regorafenib was 10.6 months (placebo: 7.8 months, HR = 0.63, *p* <0.0001). Moreover, OS subanalysis showed significantly better results for patients with a Child–Pugh score of 5 on starting sorafenib compared with patients with a score of 6. This is because patients with a score of 5 could quickly be switched from TACE to sorafenib if refractory to TACE, and then could quickly be switched from sorafenib to regorafenib if refractory to sorafenib, which will be an important strategy for improving survival going forward.

**Figure 1:**
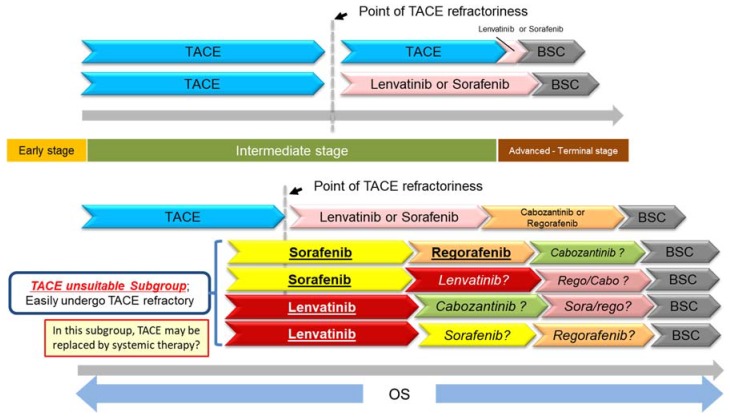
New treatment landscape in HCC. BSC: best supportive care.

The results of the RESORCE trial also showed that sorafenib–regorafenib sequential therapy yielded good OS (26 months from starting sorafenib vs. 19.2 months for placebo) [41,42]. This is an extremely important finding. This long survival time of 26 months nearly rivals conventional TACE outcomes for intermediate-stage HCC [12,42]. The only phase III prospective trial with survival times for the TACE placebo arm presented is the BRISK TA trial, which has the largest enrollment of any such trial in the world. For the above reasons, the outcomes of the placebo arm in this trial could currently be considered the global standard for TACE outcomes with no selection bias whatsoever. The patient population for this trial was 82% early/intermediate-stage (BCLC B: 59%; BCLC A: 23%; BCLC C: 17%), with only 17% of participants in the advanced stage. In contrast, the RESORCE trial enrolled 86% BCLC C advanced-stage patients. When the two cohorts are compared directly, OS is comparable between TACE and sorafenib–regorafenib sequential therapy (26.1 months vs. 26 months). It may not be appropriate to compare individual arms of completely different randomized controlled trials (RCTs), but they are placebo arms of well-designed RCTs, and thus have no selection bias. At the very least, the fact that OS is comparable between the two is very important because sorafenib-regorafenib sequential therapy was applied to a population with much more advanced disease (i.e., advanced-stage HCC). Undoubtedly the patient population is certainly highly selected, but this means that the same effect obtained with TACE in the population for which TACE is indicated can be obtained with sorafenib-regorafenib sequential therapy in patients with advanced-stage HCC. Now that the potential of sorafenib-regorafenib sequential therapy to greatly improve prognosis is clear, it may be necessary to re-evaluate the appropriate timing for starting sorafenib. The conventional practice has been to switch from TACE to systemic therapy at the point when the patient is found to be refractory to TACE, but one could envision that it may become increasingly important to identify subgroups that tend to be refractory to TACE and start systemic therapy earlier than usual in those groups (while hepatic functional reserve is still Child-Pugh 5 before they are found to be refractory to TACE) [42] (Figure 1). These patient subgroup can be categorized as “TACE unsuitable patient subpopulation”.

#### Cabozantinib: Overview of the CELESTIAL Trial

The results of this trial were presented at ASCO-GI in 2018 [33]. The study enrolled 773 patients with unresectable HCC that had progressed following at least one prior systemic chemotherapy regimen containing sorafenib from September 2013 to September 2017.

This trial showed significantly better OS in the cabozantinib arm (10.2 months, 95% CI 9.1–12.0) than in the placebo arm (8.0 months, 95% CI 9.1–12.0). The secondary endpoint, PFS, was also better in the cabozantinib arm (5.2 months, 95% CI 4.0–5.5) than the placebo arm (1.9 months, 95% CI 1.9–1.9). In addition, ORR was better in the cabozantinib arm than in the placebo arm (4% vs. 0.4%) (*p* = 0.0086). Post-trial treatment was performed for a comparably low proportion of patients in the cabozantinib and placebo arms (25% vs. 30%).

Cabozantinib and regorafenib had comparable efficacy in terms of OS, ORR, and PFS. Comparable results were obtained for patients who only received prior treatment with sorafenib.

Treatment duration with cabozantinib was 3.8 months, which was similar to that of regorafenib (3.6 months), suggesting good tolerability. Dose reduction and discontinuation due to treatment-related AEs was slightly more common in cabozantinib than in regorafenib. Specific AEs such as hand–foot skin reaction and diarrhea were more common in cabozantinib than in regorafenib, indicating that cabozantinib may be slightly more toxic [43].

#### Ramucirumab: Overview of the REACH-2 Trial

Results of the REACH-2 trial were reported at the ASCO annual meeting in June 2018 [44]. OS was 8.5 months in the ramucirumab group, and 7.3 months in the placebo group; the difference was significant (HR = 0.710, 95% CI: 0.531–0.949, *p* = 0.0199) (Table 3). Ramucirumab therapy decreased the mortality rate by 29%. In all subgroups, except the female subgroup, OS was longer in patients who received ramucirumab than those placebo, particularly in men, those with extra-hepatic metastases, and those without vascular invasion.

PFS was 2.8 months in the ramucirumab group, and 1.6 months in the placebo group; the difference was significant (HR = 0.452, 95% CI: 0.339–0.603, *p* < 0.0001). Ramucirumab-treated patients had favorable PFS in all subgroups. ORR was 4.6% (95% CI: 1.7–7.5), with no complete response (CR) cases and nine partial response (PR) cases in the ramucirumab group, and 1.1% (95% CI: 0.0–3.1) with no CR case and one PR case in the placebo group; the difference was not significant (*p* < 0.1697) due to the limited number of cases. Disease control rate (DCR) was significantly better in the ramucirumab group than in the placebo group (*p* = 0.0006); it was 59.9% (118 cases comprising 0 CR, 9 PR, and 109 SD cases) in the former group, with 38.9% (37 cases comprising no CR, 1 PR, and 36 SD cases) in the latter group.

The median dose exposure in the ramucirumab group was six (range, 3–13) cycles, while that in the placebo group was four (range, 3–6) cycles. The relative dose intensity in the former was 97.7% while that in the latter was 99.8%, indicating that ramucirumab therapy was almost uninterrupted in all cases. Ramucirumab targets a single molecule (VEGFR-2) and is likely to have fewer adverse events and favorable tolerability. Rates of study drug discontinuation due to adverse events were 10.7% and 3.2% in the ramucirumab group and the placebo group, respectively, and those of dose modification were 34.5% and 13.7% in the former group and the latter group, respectively. Grade ≥3 adverse events that occurred in ≥5% of patients were hypertension (12.7% in the ramucirumab group vs. 5.3% in the placebo group), bleeding (5.1% vs. 3.2%), and liver damage (18.3% vs. 15.8%).

REACH-2, which reexamined ramucirumab in patients with baseline AFP level ≥400 ng/mL based on the results of the preceding REACH, was a positive study confirming significantly longer OS in ramucirumab- than in placebo-treated patients. PFS and DCR were also significantly better, indicating the drug’s potency. Results regarding adverse events were similar to those shown in ramucirumab monotherapy for other indications, suggesting good tolerability in these patients. REACH-2 is an excellent trial because it is the first prospective randomized controlled biomarker-driven trial with positive outcomes. 

Kaplan–Meier survival curves showed a significant difference in survival rate between the ramucirumab group (24.5%) and the placebo group (11.3%) at 18 months (*p* = 0.0187), but not at 12 months. This can be explained by the imbalance between the two groups regarding baseline AFP level and the proportion of BCLC C. Because both were higher in the ramucirumab group than in the placebo group, the effect of ramucirumab was not apparent until the latetreatment period. Furthermore, patients with baseline AFP level ≥400 ng/mL, which is associated with poor prognosis, might have died while on placebo without having post progression treatment. Selection of subjects of the trial using the level of a biomarker (in this instance, AFP), contributed to the positive outcomes despite a very small number of patients (*n* = 292) for a clinical trial of a second-line agent. 

Comparison of the results of the placebo group with baseline AFP level ≥400 ng/mL showed that OS was 7.3 months in the REACH-2 trial, which was longer than the 4.2 months in the REACH trial. This can be explained by the imbalance in patient characteristics regarding baseline APF level, mentioned earlier. The AFP value in the placebo arm (which is not available so far) in the REACH (AFP ≥400 ng/mL) trial must have been much higher than that in the placebo arms of REACH-2 (2741) since AFP value in placebo arm in pooled data of REACH (400 ≥ng/mL) and REACH-2 was much higher, 4047.5 ng/mL (Table 4). For the same reason, the HR for OS was slightly lower in the REACH-2 trial (0.67) than in patients with AFP level ≥400 ng/mL in the REACH trial (0.71) [45].

## 3. Immune Checkpoint Inhibitors

### 3.1. Immune Checkpoints

The immune checkpoint molecule PD-1 was first discovered in 1992 by Professor Tasuku Honjo and his research team at Kyoto University, Kyoto, Japan. It was named programmed death-1 (PD-1) because the researchers were looking for molecules that induced T lymphocyte apoptosis when they discovered it [46]. It was later discovered to be a receptor that negatively regulates immune responses. The PD-1 ligands PD-L1 and PD-L2 were also discovered in 2000 [47]. It was then discovered that inhibition of this pathway can eliminate tumors by reversing the tumor’s immunosuppressive effects and restoring innate immune activity, which prompted the subsequent development of antitumor drugs exploiting that mechanism in 2002 [48]. In 1995, James Allison discovered cytotoxic T-lymphocyte-associated antigen 4 (CTLA-4) [49] and found that inhibition of its function caused tumors to disappear in mice [50]. Such molecules that regulate T lymphocyte activity are called immune checkpoint molecules, and drugs that inhibit these molecules are called immune checkpoint inhibitors. Trials investigating nivolumab and pembrolizumab as anti–PD-1 antibodies, avelumab, durvalumab, and atezolizumab as anti–PD-L1 antibodies, and ipilimumab and tremelimumab as anti–CTLA-4 antibodies for HCC are currently underway [51].

### 3.2. Nivolumab

Nivolumab is the world’s first recombinant human IgG4 monoclonal antibody against human PD-1. In a phase I/II trial in advanced HCC (Checkmate-040 trial), it yielded a response rate of 20%, including two complete responses and a disease control rate of 67%, which are extremely promising results [52] (Table 5). Another unique feature of nivolumab was that its effects persisted in responders [52]. Enrollment for the trial was expanded after that point. The updated results were presented at ASCO 2017, and the OS results of 28.6 months for first-line therapy and 15.6 months for second-line therapy were promising [53]. A phase III head-to-head trial against sorafenib is currently in progress. In light of the above results of the phase I/II trial, nivolumab was designated for priority review by the United States Food and Drug Administration (FDA) and was approved in September 2017.

### 3.3. Pembrolizumab

Pembrolizumab, like nivolumab, is a recombinant human IgG4 monoclonal antibody against human PD-1. It was investigated for HCC in a phase II trial with a similar result to that of nivolumab [54] (Table 5) and is currently being investigated in a placebo-controlled phase III trial as second-line therapy for patients who have HCC refractory to sorafenib or are intolerant to sorafenib (Table 2).

### 3.4. Other Immune Checkpoint Inhibitors

Most of PD-L1 antibodies in development have only progressed to phase I or phase II trials so far. Avelumab is being developed in combination with axitinib. Atezolizumab is being developed in combination with bevacizumab. Durvalumab is being developed for combination therapy with the anti–CTLA-4 antibody, tremelimumab [58]. However, recently these 2 latter combination therapies moved forward to phase III trials as mentioned later. Early trials of other drugs, including antibodies that inhibit the immunosuppressive checkpoint molecules TIM3 and Lag3 as well as an antibody that stimulates the immune stimulatory molecule, OX40, are also in progress.

### 3.5. Combination Therapy with Immune Checkpoint Inhibitors and Molecular Targeted Agents

Results of an open-label phase Ib trial assessing the efficacy and safety of lenvatinib plus pembrolizumab were presented at ESMO 2016. In this trial, which enrolled 13 patients with solid cancers, the therapy yielded a remarkable antitumor effect as demonstrated by the response rate of 69.2% (PR: *n* = 9, SD: *n* = 4) and disease control rate of 100% [59]. Though treatment outcomes for immune checkpoint inhibitors alone have certainly garnered attention, there has been particular interest in the efficacy of combination therapy with molecular targeted drugs. Trials of immune checkpoint therapy with curative treatment for HCC have also been started (Table 1). A phase III head-to-head trial of atezolizumab plus bevacizumab against sorafenib is currently ongoing (Table 2) since very high response rate (61% per RECIST 1.1 by investigator assessment) was shown at ASCO 2018 [30]. However, updated results, presented on 21 October at ESMO 2018 showed a decreased response rate (32%) with this combination therapy [56] (Table 5). Other phase 1b combination therapies, such as pembrolizumab plus lenvatinib [55] or SHR 1210 plus aptinib [57] are ongoing. Also, a phase III head-to-head trial of durvalumab plus tremelimumab against sorafenib is ongoing (Table 2). These combination therapy approaches are extremely promising because combining the two drugs produces not just an additive effect but rather a synergistic effect against the immunosuppressive tumor microenvironment [60,61].

## 4. Conclusions

This was a review of systemic therapy for HCC. Lenvatinib and regorafenib are now available in addition to sorafenib as molecular targeted agents for the treatment of HCC. Cabozantinib and ramucirumab may also be approved in 2019. The increase in the number of molecular targeted therapy options for HCC will benefit many patients, but will probably make drug selection and sequences challenging. Combination therapy using targeted treatments with immune checkpoint inhibitors such as atezolizumaband pembrolizumab is expected to yield even better effects when these drugs eventually become available. These new drugs or combination therapy may benefit a wide range of patients from the early, intermediate stage of HCC as an adjuvant use, and advanced stages of HCC, therefore progress in their development is highly anticipated.

## Figures and Tables

**Table 1:** Randomized phase II, phase III clinical trials of early/intermediate stage hepatocellular carcinoma (HCC).

Target Population		Design	Trial Name	Result	Presentation	Publication	First Author
Early	Adjuvant (prevention of recurrence)	1. Vitamin K2 vs. Placebo 2. Peretinoin vs. Placebo 3. Sorafenib vs. Placebo 4. Peretinoin vs. Placebo 5. Nivolumab vs. Placebo	NIK-333 STORM NIK-333/K-333 CheckMate 9DX	Negative Negative Negative Ongoing Ongoing	ASCO 2010 ASCO 2014	*Hepatology* 2011 [6] *J Gastroenterol* 2014 [8] *Lancet Oncology* 2015 [7]	Yoshida H Okita K Bruix J
	Improvement of RFA	1. RFA +/− LTLD 2. RFA +/− LTLD	HEAT OPTIMA	Negative	ILCA 2013	*Clin Cancer Res* 2017 [9]	Tak WY
Intermediate	Improvement of TACE	1. TACE +/− Sorafenib 2. TACE +/− Sorafenib 3. TACE +/− Brivanib 4. TACE +/− Orantinib 5. TACE +/− Sorafenib 6. TACE +/− Sorafenib	Post-TACE SPACE (PhII) BRISK-TA ORIENTAL TACE-2 TACTICS (Ph II)	Negative Negative Negative Negative Negative Positive	ASCO-GI 2010 ASCO-GI 2012 ILCA 2013 EASL 2015 ASCO 2016 ASCO-GI 2018 [2]	*Eur J Cancer* 2011 [10] *J Hepatol* 2016 [11] *Hepatology* 2014 [12] *Lancet Gastroenterol Hepatol* 2018 [13] *Lancet Gastroenterol Hepatol* 2017 [14]	Kudo M Lencioni R Kudo M Kudo M Meyer T Kudo M

Red: positive trial; blue: ongoing trial; black: negative trials. LTLD: lyso-thermosensitive liposomal doxorubicin; RFA: radiofrequency ablation; TACE: transcatheter arterial chemoembolization.

**Table 2:** Phase III clinical trials of advanced-stage HCC.

Target Population		Design	Trial Name	Result	Presentation	Publication	First Author
Advanced	First line	1. Sorafenib vs. Sunitinib 2. Sorafenib +/− Erlotinib 3. Sorafenib vs. Brivanib 4. Sorafenib vs. Linifanib 5. Sorafenib +/− Doxorubicin 6. Sorafenib +/− HAIC 7. Sorafenib +/− Y90 8. Sorafenib +/− Y90 9. Sorafenib vs. Lenvatinib 10. Sorafenib vs. Nivolumab 11. Sorafenib vs. Durvalumab + Tremelimumab vs. Durva 12. Sorafenib vs. Atezolizumab + Bevacizumab 13. Sorafenib vs. Tislelizumab	SUN1170 SEARCH BRISK-FL LiGHT CALGB 80802 SILIUS SARAH SIRveNIB REFLECT CheckMate-459 HIMALAYA IMbrave150	Negative Negative Negative Negative Negative Negative Negative Negative Positive Ongoing Ongoing Ongoing Ongoing	ASCO 2011 ESMO 2012 AASLD 2012 ASCO-GI 2013 ASCO-GI 2016 EASL 2016 EASL 2017 ASCO 2017 ASCO 2017	*JCO* 2013 [15] *JCO* 2015 [18] *JCO* 2013 [16] *JCO* 2015 [17] *Lancet GH* 2018 [21] *Lancet-O* 2017 [22] *JCO* 2018 [23] *Lancet* 2018 [25]	Cheng AL Zhu AX Johnson PJ Cainap C Kudo M Vilgrain V Chow P Kudo M
	Second line	1. Brivanib vs. Placebo 2. Everolimus vs. Placebo 3. Ramucirumab vs. Placebo 4. S-1 vs. Placebo 5. ADI-PEG 20 vs. Placebo 6. Regorafenib vs. Placebo 7. Tivantinib vs. Placebo 8. Tivantinib vs. Placebo 9. DT vs. Placebo 10. Cabozantinib vs. Placebo 11. Ramucirumab vs. Placebo 12. Pembrolizumab vs. Placebo	BRISK-PS EVOLVE-1 REACH S-CUBE NA RESORCE METIV-HCC JET-HCC ReLive CELESTIAL REACH-2 KEYNOTE-240	Negative Negative Negative Negative Negative Positive Negative Negative Negative Positive Positive Ongoing	EASL 2012 ASCO-GI 2014 ESMO 2014 ASCO 2015 ASCO 2016 WCGC 2016 ASCO 2017 ESMO 2017 ILCA 2017 ASCO-GI 2018 ASCO 2018	*JCO* 2013 [26] *JAMA* 2014 [27] *Lancet-O* 2015 [28] *Lancet GH* 2017 [29] *Ann Oncol* 2018 [30] *Lancet* 2017 [31] *Lancet-O* 2018 [32] *NEJM* 2018 [33]	Llovet JM Zhu AX Zhu AX Kudo M Abou-Alfa G Bruix J Rimassa L Abou-Alfa G Zhu AX

Red: positive trials; blue: ongoing trials; black: negative trials. HAIC: hepatic arterial infusion chemotherapy; ADI-PEG 20: arginine deiminase-conjugated with polyethylene glycol; DT: doxorubicin-loaded nanoparticles.

**Table 3:** Results of the REACH-2 Trial.

Efficacy and Tolerability	Ramucirumab (*n* = 197)	Placebo (*n* = 95)	HR (95% CI)	*p*-Value
mOS	8.5 m	7.3 m	0.710	0.0199
mPFS	2.8 m	1.6 m	0.452	0.0001
ORR	4.6%	1.1%	-	0.1967
Relative dose intensity	97.9%	99.8%	-	-
Discontinuation due to TEAE	10.7%	3.2%	-	-
Dose adjustment due to AE	34.5%	13.7%	-	-

OS: Overall survival; PFS: progression free survival; TEAE: Treatment-emergent adverse event; ORR: objective response rate; AE: adverse event. Cited and modified from ref. [45].

**Table 4:** Comparison between REACH (AFP ≥ 400 ng/mL), REACH-2, and pooled data. OS: overall survival; AFP: alpha-fetoprotein.

Study Name	REACH (AFP ≥ 400 ng/mL) (*n* = 250)		REACH-2 (*n* = 292)		Pooled REACH-2/REACH (AFP ≥ 400 ng/mL) (*n* = 542)	
Efficacy and AFP	Ram	Placebo	Ram	Placebo	Ram	Placebo
OS (month) (median)	7.8	4.2	8.5	7.3	8.1	5.0
HR (95% CI)	0.674 (0.508, 0.895)		0.710 (0.531, 0.949)		0.694 (0.571, 0.842)	
*p*-value	0.0059		0.0199		0.0002	
AFP (ng/mL) (median)	N/A	N/A	3920	2741	4104.6	4047.5

N/A: Not available.

**Table 5:** Results of immune checkpoint inhibitors and combination therapy.

Efficacy	Nivolumab [52]	Pembrolizumab [54]	Pembrolizumab Plus Lenvatinib [55]	Atezolizumab Plus Bevacizumab [56]	SHR-1210 Plus Apatinib [57]	Durvalumab Plus Tremelimumab [58]
	(*n* = 214)	(*n* = 104)	(*n* = 30)	(*n* = 77)	(*n* = 18)	(*n* = 40)
ORR (%, 95% CI)	20 (15–26)	17 (11–26)	42.3 (23.4–63.1)	32	38.9	25
DCR (%, 95% CI)	64 (58–71)	62 (52–71)	100	77	83.3	57.5 (>16 week)
PFS (Month, 95% CI)	4.0 (2.9–5.4)	4.9 (3.4–7.2)	9.7 (5.6–NE)	14.9 (0.5–21.5)	7.2 (2.6–NE)	NA
OS (Month, 95% CI)	NR (9M OS, 74%)	12.9 (9.7–15.5)	NR	NR	NR	NA
DOR (Month)	9.9 (8.3–NE)	≤9 (77%)	NE	≥12 (26%)	NE	NA

ORR: objective response rate; DCR: disease control rate; PFS: progression free survival; OS: overall survival; DOR: duration of response; NR: not reached; NE: not estimable; NA: not available.
